# GWIDD: a comprehensive resource for genome-wide structural modeling of protein-protein interactions

**DOI:** 10.1186/1479-7364-6-7

**Published:** 2012-07-11

**Authors:** Petras J Kundrotas, Zhengwei Zhu, Ilya A Vakser

**Affiliations:** 1 Center for Bioinformatics, The University of Kansas, 2030 Becker Dr., Lawrence, KS, 66047, USA; 2 Department of Molecular Biosciences, The University of Kansas, 2030 Becker Dr., Lawrence, KS, 66047, USA; 3 Current address: Department of Genetics, Room 716B, Abramson Research Center, University of Pennsylvania, 3615 Civic Center Blvd., Philadelphia, PA, 19104, USA

**Keywords:** Protein-protein interactions, Structural modeling, Protein docking, Structural genomics, Interactome

## Abstract

Protein-protein interactions are a key component of life processes. The knowledge of the three-dimensional structure of these interactions is important for understanding protein function. Genome-Wide Docking Database (http://gwidd.bioinformatics.ku.edu) offers an extensive source of data for structural studies of protein-protein complexes on genome scale. The current release of the database combines the available experimental data on the structure and characteristics of protein interactions with structural modeling of protein complexes for 771 organisms spanned over the entire universe of life from viruses to humans. The interactions are stored in a relational database with user-friendly interface that includes various search options. The search results can be interactively previewed; the structures, downloaded, along with the interaction characteristics.

## Introduction

Proteins function by interacting with other biologically relevant molecules. Understanding the mechanisms of protein-protein interactions (PPI) is essential for studying life processes at the molecular level. Genome sequencing provided a vast amount of information on proteins at the sequence level. Currently, efforts focus on the function assignment of these proteins based on their three-dimensional (3D) structures and interactions. Interaction maps for specific organisms and biochemical pathways need to be complemented by the structural information. Experimental techniques are limited in their ability to produce the structures on the genome scale. Thus, computational methods are essential for this task [1].

Structural modeling of PPI has its origins in *ab initio* techniques based on shape and physicochemical complementarity. More recent approaches take advantage of statistical potentials and machine learning [2,3]. Despite progress in development of such template-free algorithms, their accuracy in the high-throughput structure determination is limited.

Rapidly increasing amount of data on PPI makes possible application of the template-based methods. Such approaches are based on the observation that monomers with similar sequences and/or structures, generally, have similar binding modes. Several groups assessed the quality of PPI modeling based on sequence alignment to complexes with known structure [4-9]. Studies showed that the majority of such homology-docking models are of acceptable and medium quality, according to the established criteria [3]. An alternative template-based approach takes advantage of the structural similarity between the target and the template complexes [10-13].

The progress in 3D modeling of PPI is reflected in the Genome-Wide Docking Database (GWIDD) [14], which provides annotated collection of experimental and modeled PPI structures from the entire universe of life spanning from viruses to humans. The resource has user-friendly search interface, providing preview and download options for experimental and modeled PPI structures.

### Database design

**Figure 1:**
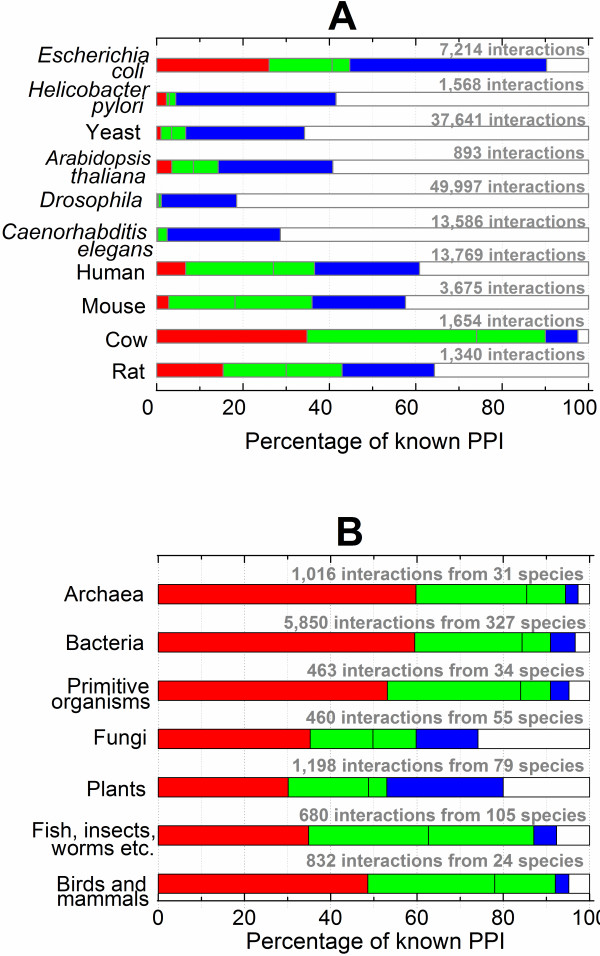
**Structural coverage of different genomes in GWIDD.** X-ray structures of complexes are in *red*, sequence-based models of complexes are in *green*, and interactions with structural models of the monomers are in *blue*. (**A**) Ten genomes with the largest number of known PPI. (**B**) The rest of the genomes (the data from A excluded).

GWIDD imports PPI from external sources, including the last free release of BIND [15] and DIP [16,17]. Currently, we are working on interfacing GWIDD with MINT [18], BioGRID [19], and IntAct [20]. To provide the structures to PPI, the following scheme is utilized. If the complex is found in the Protein Data Bank (PDB), the X-ray structure is used, and no modeling is performed (10,924 GWIDD entries). Otherwise, a search for a pair of homologous sequences from complexes with known structure is performed, and the model is built by homology docking [6,7]. Statistical significance of the sequence alignments is assigned [7], with an additional requirement that both alignments contain at least 80% of the target sequences. This provides structures for 12,646 PPI. For the interactions not covered by these two steps, the interacting monomers are modeled independently by homology modeling, with subsequent docking of the models by structural alignment [12]. Incorporation of the structural alignment predictions (28,811 entries) into GWIDD is currently in progress (the structures are available from the authors by request). The graphical summary of the GWIDD coverage of genomes is in Figure 1.

### User interface

**Figure 2:**
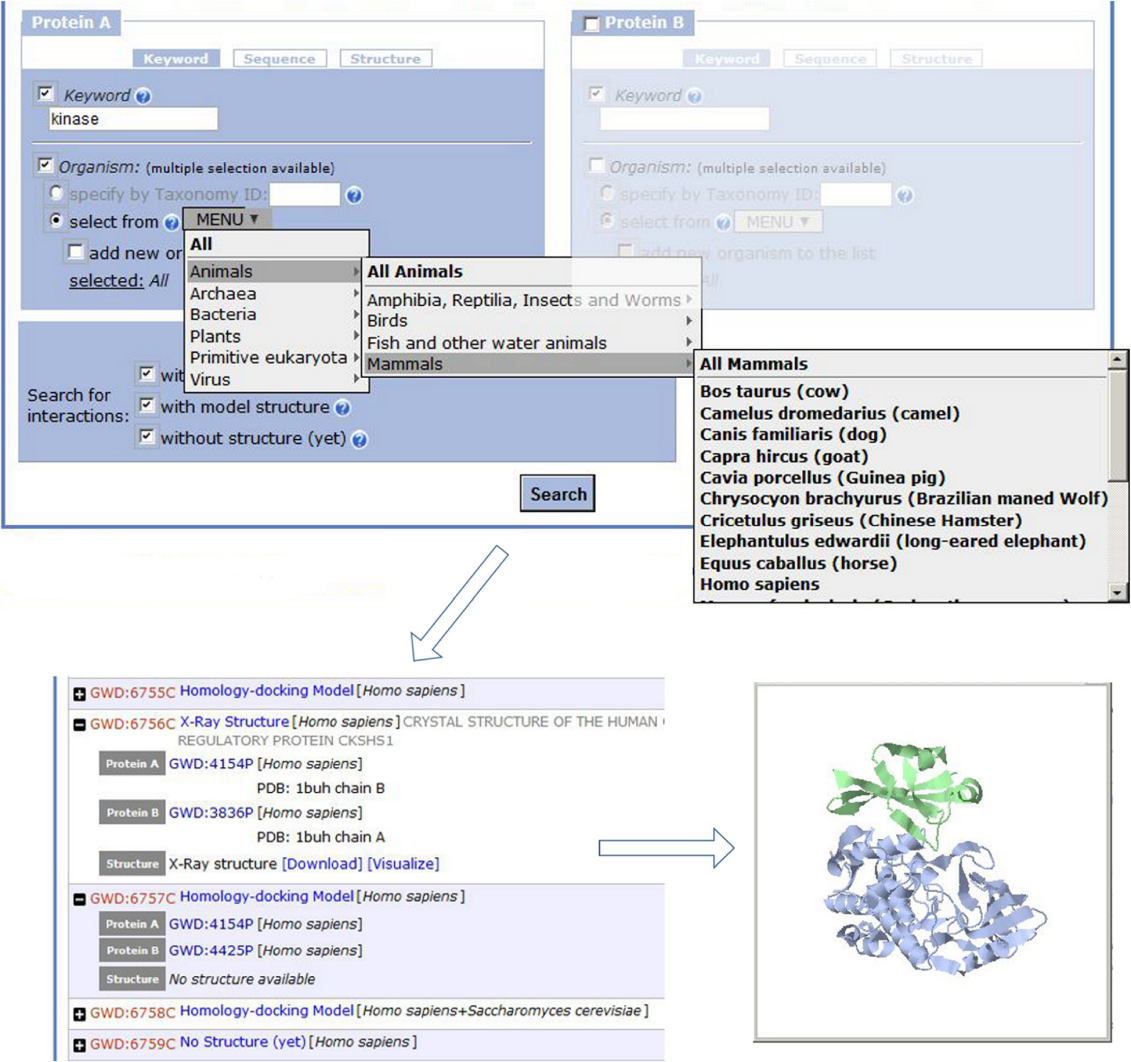
Example of a search.

The database (http://gwidd.bioinformatics.ku.edu) user interface (Figure 2) offers search by keywords, sequences (explicit input or upload in FASTA format), or structures (upload in PDB format), for one or both interacting proteins. The search by keywords can be performed using any word in the protein description (name of organism, cellular location, biological function etc.) or by selection from drop-down menus that are listing organisms currently in GWIDD. Repeated selection of the box ‘Add another organism to the list’ allows expansion of the search to several organisms. An option for search by standard taxonomy identification (ID) with link to taxonomy database http://www.uniprot.org is also provided. In case of input PDB file, the sequence is extracted from SEQRES tags or, if the SEQRES is not available, from ATOM tags of C^α^ atoms. The sequences from different sources can differ in length even for the same protein (e.g., due to unresolved fragments of the X-ray structure). Thus, advanced sequence search options are available. Figure 2 shows an example of search by organism.

The user can enable the second half of the search interface if information related to the interaction partner is available (‘protein B,’ Figure 2). The search results can be filtered by the structure availability (experimental, modeled, or no structures). Online help is provided in pop-up windows. The search result screen displays all interactions in the database satisfying the input search criteria in the form of an expandable list of GWIDD interaction IDs. For the homology-docking models, the alignments used to build the model are provided, and the model quality is assessed by the sequence identity criteria [5]. Links are provided to download the PDB-format files, along with the text file containing relevant information. Visualization screen is available to display the structures by different interactive representations. A link is provided to download the entire set of sequence-homology models in one gzipped archive.

### Implementation

GWIDD unifies different external PPI data formats into a single data set, removing redundancy and retaining common data fields for all the sources. The interaction data are stored in a relational database, except for large files, such as structure coordinates, which are stored directly in the file system and are linked from the relational database. The web interface is implemented on the Linux-Apache-PostgreSQL-PHP software stack. Web user interface is built using hypertext preprocessor (PHP) and jQuery library, where PHP is for web presentation and logic as well as back-end database access; jQuery is responsible for AJAX and other JavaScript-based dynamic features. Visualization of protein structures is implemented in Jmol (http://www.jmol.org). Homology docking was performed by NEST [21], BLAST [22], and in-house profile-to-profile alignment program. The procedures are joined by Python scripts.

### Future directions

GWIDD development will incorporate other structural modeling techniques, such as multi-template/threading modeling of interacting proteins, partial structural alignment [12], and template-free docking by GRAMM [23-25]. A major expansion of GWIDD will be the incorporation of new PPI sources from other publicly available PPI databases. Large-scale systematic benchmarking of the high-through put modeling will be used to assign a confidence score to the modeled structures.

## Competing interests

The authors declare that they have no competing interests.

## Authors’ contributions

ZZ performed calculations and implemented the web interface. PJK developed calculation pipelines, designed the web interface, analyzed data, and drafted the manuscript. IAV designed the research, analyzed data, and wrote the paper. All authors read and approved the final manuscript.
